# Study of Osteoarthritis-Related Hub Genes Based on Bioinformatics Analysis

**DOI:** 10.1155/2020/2379280

**Published:** 2020-08-05

**Authors:** Zhengqing Zhu, Lei Zhong, Ronghang Li, Yuzhe Liu, Xiangrun Chen, Zhaoyan Li, Lanfeng Huang

**Affiliations:** Departments of Orthopaedics, The Second Hospital of Jilin University, Changchun, Jilin, China

## Abstract

Osteoarthritis (OA) is a common cause of morbidity and disability worldwide. However, the pathogenesis of OA is unclear. Therefore, this study was conducted to characterize the pathogenesis and implicated genes of OA. The gene expression profiles of GSE82107 and GSE55235 were downloaded from the Gene Expression Omnibus database. Altogether, 173 differentially expressed genes including 68 upregulated genes and 105 downregulated genes in patients with OA were selected based on the criteria of ∣log fold‐change | >1 and an adjusted *p* value < 0.05. Protein-protein interaction network analysis showed that *FN1*, *COL1A1*, *IGF1*, *SPP1*, *TIMP1*, *BGN*, *COL5A1*, *MMP13*, *CLU*, and *SDC1* are the top ten genes most closely related to OA. Quantitative reverse transcription-polymerase chain reaction showed that the expression levels of *COL1A1*, *COL5A1*, *TIMP1*, *MMP13*, and *SDC1* were significantly increased in OA. This study provides clues for the molecular mechanism and specific biomarkers of OA.

## 1. Introduction

Osteoarthritis (OA) is a common orthopedic disease that occurs in middle-aged and elderly people [1, 2]. OA is characterized by joint degeneration, with the main clinical manifestations being pain, swelling, and limited movement of the involved joints. Its etiology is related to joint injury, joint or limb dysplasia, age, obesity, and genetic factors. OA is among the greatest causes of morbidity and disability worldwide [3]. According to the estimation, it is estimated that approximately 31 million people in the United States are affected [4]. Local or systemic inflammation is considered to play a key role in the progress of OA, particularly in the early stage of disease [5]. Although abnormal gene expression in OA synovial cells has been widely reported in human and animal studies, altered gene expression and regulation in OA synovial cells remain unclear [6].

In recent years, a lot of research had been conducted to explore the molecular characteristics of OA. High-throughput microarray methods have attracted attention and are widely used in fields such as medical oncology ranging from diagnosis to treatment and prediction of the pathogenesis and prognosis of patients and for identifying drug targets [7–9]. Epigenetic regulation of gene expression in OA progression has recently been reported, and bioinformatics and microarray technologies have been extensively conducted to screen gene signaling pathways of OA [10, 11], providing strong theoretical support for the diagnosis as well as the treatment of OA [12, 13]. The roles of some differentially expressed genes (DEGs) in OA were studied by bioinformatics analysis and microscopy, enabling studies of the complex process of OA occurrence and development [14]. However, microarray methods are limited by the small number of samples collected, measurement errors, and inadequate information collection. The integrative analysis of multiple factors in OA remains challenging, and the exact pathogenesis of this condition remains unclear.

Despite these limitations, microarray systems are still efficient tools for detecting gene expression, identifying biomarkers, and evaluating epigenetic variation [15]. Network modeling of protein-protein interactions (PPIs) is a new technology for studying diseases and identifying disease-related gene targets [16]. To further explore DEGs and their molecular biological roles in OA, we have downloaded raw microarray data (GSE82107, GSE55235) from the Gene Expression Omnibus (GEO). DEGs between osteoarthritis synovium and normal controls were screened out by a bioinformatics method. A PPI network was built to enrich the protein domain of genes in the PPI network module and screen for hub genes related to OA. Significant DEGs between patients with OA and normal subjects may play an important part in OA development and progression. Additionally, the understanding of the underlying molecular mechanism of the pathogenesis of OA may be improved by using this method, revealing new therapeutic approaches for the epigenetic regulation of OA. In this research, we intended to explore the pathogenesis of OA through gene expression analysis to identify new potential biomarkers.

## 2. Materials and Methods

### 2.1. Microarray Data Source

The GEO dataset is a public functional genomics data repository that stores public gene expression datasets and platform records [17]. The gene expression profiles of GSE82107 and GSE55235 were downloaded from the GEO database. The GSE82107 dataset was obtained by using the GPL570 platform (Affymetrix Human Genome U133 Plus 2.0 Array, Santa Clara, CA, USA); the microarray data of GSE82107 contained 20 synovium samples from patients with OA and 17 knee synovium samples from normal subjects [18]. The GSE55235 dataset was based on the GPL96 platform (Affymetrix Human Genome U133A Array), and the microarray data of GSE55235 contained 10 OA patients synovium and 10 normal knee synovium [19].

### 2.2. Identification of Differentially Expressed Genes

Raw data were corrected using the RMA method before analyzing DEGs in OA, and we used a limma package in R software to screen for DEGs between the synovium of patients with OA and normal knee synovium [20]. DEGs were screened according to ∣log fold‐change (FC) | >1 and an adjusted *P* value < 0.05. Common DEGs in the GSE82107 and GSE55235 datasets were screened with FunRich software [21].

### 2.3. GO and Pathway Enrichment Analysis

To better understand the pathways and signal transduction processes involving DEGs in disease processes, the online DAVID bioinformatics database was used for GO and KEGG pathway analysis, and a gene count > 2 and *P* < 0.05 were considered to indicate significant results [22]. The GO database is the world's largest source of information on gene function, with enrichment analysis including the molecular function (MF), cellular component (CC), and categories of biological processes (BP). [23].

### 2.4. PPI Network Construction

The STRING database is used to integrate all publicly available sources of PPI information and is also used to calculate predictions based on PPI analysis [24]. We utilized the STRING database to structure a PPI network of DEGs. An interaction score > 0.400 was selected as a significant threshold. We led the raw data in Cytoscape software and used the cytoHubba plugin to build a subnetwork of PPIs and screened hub genes [25].

### 2.5. Case and Control Groups

Our research was approved by the ethics committee of the Second Hospital of Jilin University (Ethics number 2018-292). Ten patients with OA and ten patients with meniscus tears were included in this study, and all patients signed informed consent. We collected OA synovial tissue from patients undergoing total knee arthroplasty at the Jilin University Second Hospital, and normal synovial tissue was collected from patients undergoing arthroscopic surgery at the Jilin University Second Hospital.

### 2.6. Validation of Gene Expression

Real-time PCR was worked to validate the top ten hub genes, and total RNA was extracted from osteoarthritic and control synovial tissues by utilizing TRIzol reagent (Invitrogen, Carlsbad, CA, USA) followed by reverse transcription into cDNA. The mRNA expression levels were further assessed by quantitative reverse transcription- (qRT-) PCR using the QuantStudio™ 7 Flex real-time PCR system (Applied Biosystems, Foster City, CA, USA). These primers were designed with primer premier 6.0 software (Table 1). All samples were standardized to GAPDH expression; also, the experiment was repeated three times. The relative abundance of genes was calculated using the 2^-*ΔΔ*Ct^ method, and data were analyzed with GraphPad software (GraphPad, Inc., La Jolla, CA, USA). The *P* value < 0.05 was considered significant. The Pearson correlation coefficient was used to examine the relationship between key genes.

## 3. Results

### 3.1. Identification of DEGs in OA

**Figure 1:**
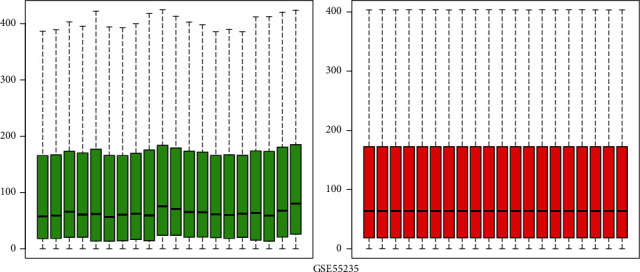
Boxplots of GSE55235 data prior to and following normalization. The red box plots represent the normalized data, and the green box plots represent the data before normalization.

**Figure 2:**
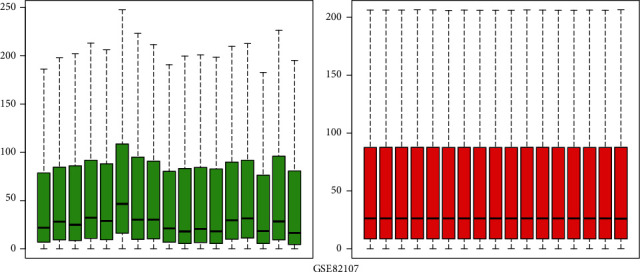
Boxplots of GSE82107 data prior to and following normalization. The red box plots represent the normalized data, and the green box plots represent the data before normalization.

**Figure 3:**
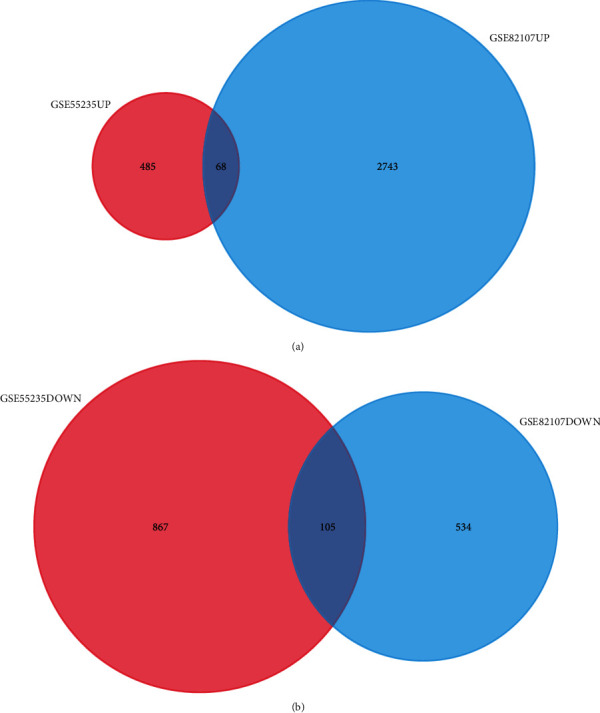
The Venn analysis of upregulated and downregulated DEGs. (a) The Venn analysis of upregulated DEGs showed there are 68 DEGs coexpressed in two gene profiles. (b) The Venn analysis of downregulated DEGs presented there are 105 DEGs that were coexpressed in two gene profiles.

We evaluated 10 patients with OA and 10 healthy control patients. After gene expression data processing and normalizing, we utilized the limma package in R software to screen for DEGs in each GEO dataset. The GSE5235 and GSE82107 datasets were standardized, and the results are shown in Figures 1 and 2. By using ∣logFC | >1 and adjusted *P* value < 0.05 as thresholds, we determined 553 upregulated genes and 972 downregulated genes in GSE55235 and also 2811 upregulated genes and 639 downregulated genes in GSE82107. Based on overlapping results in the Venn diagrams (Figure 3), 173 genes were identified; 68 genes were upregulated, and the other 105 genes were downregulated.

### 3.2. GO and KEGG Pathway Enrichment Analyses

**Figure 4:**
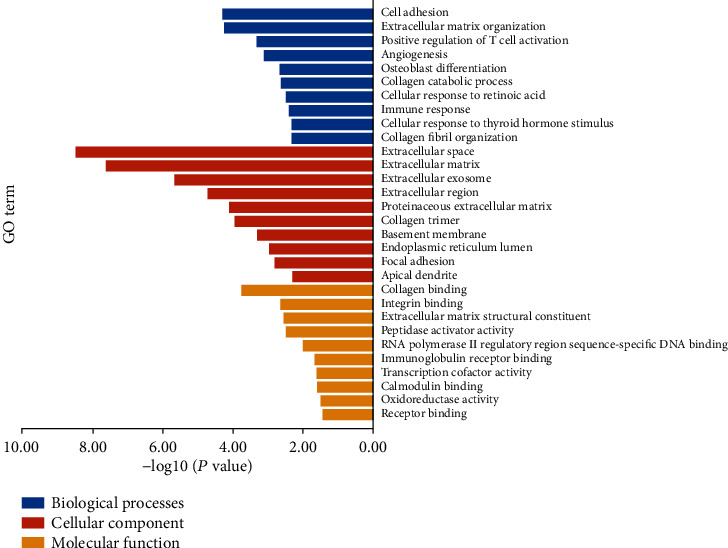
According to gene ontology analysis, DEGs can be divided into three categories: molecular function, biological process, and cellular component.

**Figure 5:**
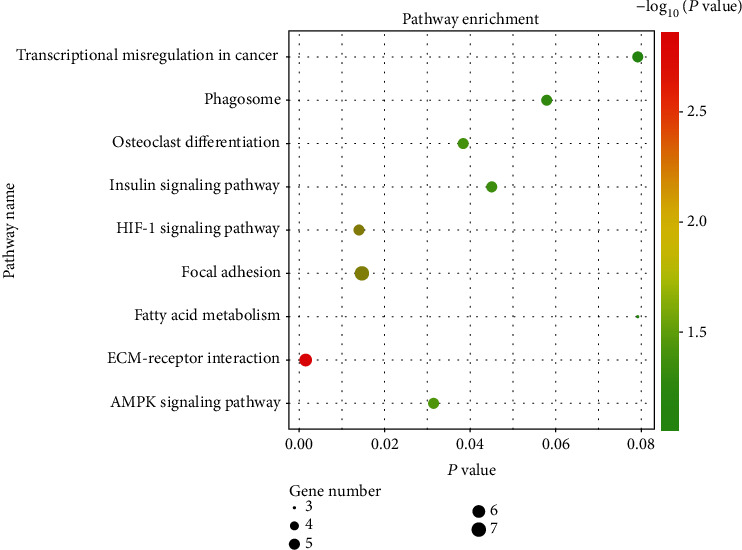
Kyoto Encyclopedia of Genes and Genomes (KEGG) enrichment analysis of the pathways. The size of the black spots stands for the gene number; the gradual color stands for the *P* value.

We conducted enrichment analysis to assess the biological processes and pathways within OA (an interaction score > 0.400 was selected as a significance). The GO enrichment analysis results were divided into three functional categories, including MF, CC, and BP. For BP, the DEGs were enriched in cell adhesion, immune response, and osteoblast differentiation (Table 2). In the CC group, the DEGs were majorly enriched in the extracellular space, extracellular matrix, and extracellular exosome. In the MF group, DEGs were majorly enriched in the collagen binding, integrin binding, and extracellular matrix structural constituent (Figure 4). We used the DAVID online database to perform DEG pathway enrichment analysis. KEGG analysis showed that DEGs play a role in regulating synovial inflammation in OA through various pathways including the AMPK signaling pathway, osteoclast differentiation, insulin signaling pathway, autophagy, ECM-receptor interaction, and HIF-1 signaling pathway (Figure 5, Table 3).

### 3.3. Identification of Hub Genes

**Figure 6:**
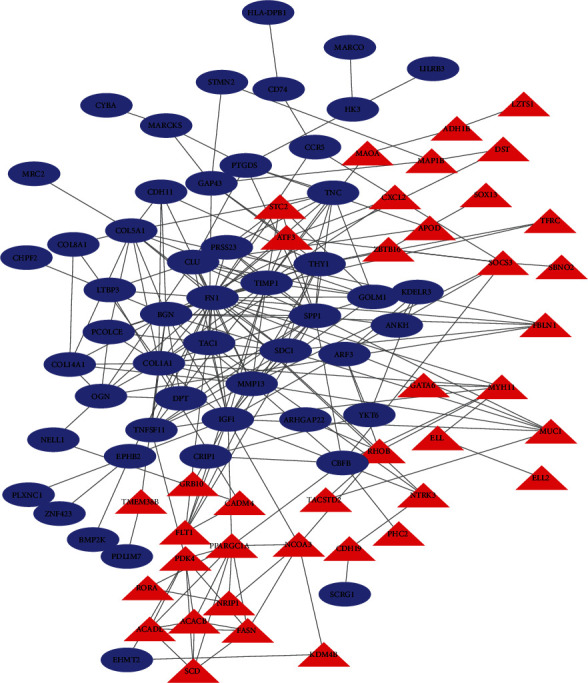
PPI network constructed with the downregulated and upregulated DEGs. Blue nodes represent upregulated genes, and red nodes represent downregulated genes. The size of the triangles and circles represents the degree value.

**Figure 7:**
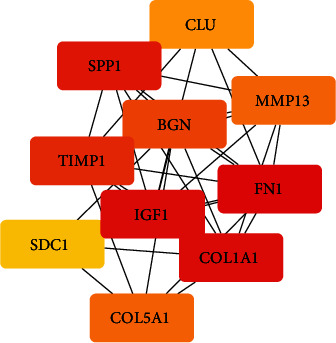
The 10 hub genes showed significant interactions, and these genes were validated by qRT-PCR. (The color of the boxes is the weight of hub genes. From red to yellow, red indicates a higher degree of association.)

To further examine the hub genes involved in the development of OA, we used the STRING database to evaluate the interaction between genes. We built a PPI network by using Cytoscape software and some data from the STRING database. To further analyze protein interactions, we evaluated the betweenness centrality and degree using the plugin cytoHubba in Cytoscape software (Figure 6). Hub genes with betweenness centrality and degree indicate that these genes play a key role in this network. The 10 hub genes showing significant interactions were *FN1*, *COL1A1*, *IGF1*, *SPP1*, *TIMP1*, *BGN*, *COL5A1*, *MMP13*, *CLU*, and *SDC1* (Figure 7).

### 3.4. Validation of Hub Genes

**Figure 8:**
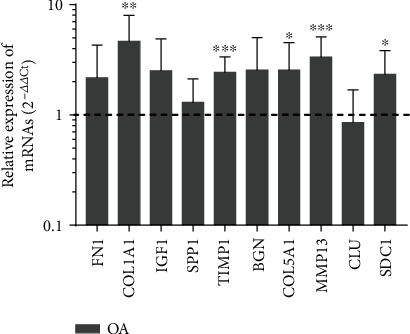
Validation of the top10 hub genes by qRT-PCR between the OA group (*n* = 10) and the control group (*n* = 10). The dotted line at ordinate 1 represented the relative mRNA expression of the control group. All samples were normalized to the expression of GAPDH, and the relative expression levels of each gene were analyzed using the 2^−*ΔΔ*Ct^ method. ^∗^*P* < 0.05, ^∗∗^*P* < 0.01, and ^∗∗∗^*P* < 0.001.

To check the data analysis results, qRT-PCR was used to detect the expression level of the first 10 hub genes in the OA synovium of the knee joint and normal control groups. Statistical analysis proved that *ADIPOQ*, *IL6*, and *CXCR1* were obviously raised in the synovium of OA samples (*P* < 0.05) (Figure 8). All validations are consistent with the results of this research.

## 4. Discussion

OA is a complex disease caused by genetic and environmental factors. This condition affects more than 40% of 70 year olds and is the main cause of loss of body movement and pain [26]. OA is characterized by several factors, including the loss of articular cartilage, hyperosteogeny, and synovitis. The combined effects of mechanical and biochemical factors are implicated in OA development. The rate of OA increases with age and mechanical wear on the joints. However, the exact pathogenesis of OA is unclear, and there are no effective treatment methods; joint replacement surgery is the last resort for treating OA [27].

Gene chip and high-throughput sequencing technologies can be used for detecting the gene expression, microRNA, long noncoding RNA, and DNA methylation to explore genetic alterations in disease [28]. Microarray techniques have also been widely used to predict potential target genes for OA [7, 29]. To decrease the number of false-positive results, we performed functional enrichment and network analysis of the DEGs. A total of 173 genes were identified, with 68 genes upregulated and 105 genes downregulated.

Using the KEGG signal pathway analysis method to analyze the enrichment of DEGs. Based on the string online database, a PPI network is built by using the software of Cytoscape, and 10 most closely related genes were screened out by the degree analysis method of the cytoHubba plugin. The following genes were highly expressed: *FN1*, *COL1A1*, *IGF1*, *SPP1*, *TIMP1*, *BGN*, *COL5A1*, *MMP13*, *CLU*, and *SDC1*. To validate these findings, the qRT-PCR results confirmed that the expression levels of *COL1A1*, *COL5A1*, *TIMP1*, *MMP13*, and *SDC1* were obviously increased in OA samples (*P* < 0.05).

The main organic component of bone is collagen type I, which consists of one pro-alpha2 (I) chain encoded by the collagen type I alpha 2 chain gene (*COL1A2*) and two pro-alpha1 (I) chains encoded by two the collagen type I alpha 1 chain genes (*COL1A1*) [30]. COL1A1 is one of several collagens showing rich differences in RNA and protein levels. Collagens are the major structural components of the cartilage. Collagen imbalance plays an important pathogenic role in OA [31, 32]. Type I collagen is the most abundant collagen in scar tissue and is the final product of tissue healing. A recent study showed that COL1A1 was upregulated in the synovium of patients with advanced OA as well as in the synovium of OA-induced mice and human fibroblasts stimulated by transforming growth factor-*β* (TGF-*β*) [33]. According to previous studies, collagen type I is rare in normal chondrocytes but is highly expressed in OA cartilage, especially in the late stage of OA [34]. The collagen type I alpha 5 chain gene (COL5A1) encodes the *α*1 chain of type V collagen and is small fibrous collagen found in ligaments, tendons, and other tissues [35]. Type V collagen, encoded by COL5A1, is a type I collagen present in tissues containing type I collagen and may play an important role in regulating the assembly of heteromorphic fibers composed of type I as well as type V collagen [36, 37]. Because these genes were found to be upregulated in OA patients, collagen may play a key role in the pathogenesis of OA.

It has been established that the aging of chondrocytes in elderly patients promotes the development of OA [38]. In this case, aging and pathological cells still survive but show changes in the matrix metalloproteinase (MMP) expression profile. MMPs are known to be involved in cartilage destruction and inflammation in the joint [39]. MMPs belong to a protease family called metzincin superfamily, which contains 23 core members and more than 30 extended members [40]. These enzymes participated in all kinds of physiological and pathological processes in life activities, such as morphogenesis, tissue repair, wound healing, and postinjury remodeling, as well as the progression of OA, chronic tissue ulcers, cancer, and arthritis [41]. MMP13 is a member of the MMP family. Due to its high expression in human OA cartilage and the ability to degrade type II collagen fibers, MMP13 is supposed to the main collagenase involved in the growth of OA [42–44]. In the past two decades, studies have shown that MMP13 is significant in the degradation of cartilage aggregation proteins and is closely related to the senescence of chondrocytes in the pathogenesis of OA [45, 46]. Therefore, MMP13 is considered a promising target for OA therapy [47]. The tissue inhibitor of metalloproteinase (TIMP) proteins forms a family of natural inhibitors of MMPs. This family consists of four members: TIMP 1, TIMP 2, TIMP3, and TIMP 4 [48, 49]. Because of the strong binding affinity between TIMP1 and MMP13, TIMP1 is a functional regulator of MMP13-related chondrocyte aging and cartilage matrix changes. Therefore, the improvement of TIMP1 activity is considered to be an effective way to treat OA [50].

Syndecan 1 (SDC1) protein is a transmembrane (type I) heparin sulfate proteoglycan, which is a member of the syndecan proteoglycan family [51]. SDC1 protein has several molecular roles in proliferation, migration, and cell-matrix interactions through its extracellular matrix protein receptor. In the early stage of cartilage degeneration in OA, the expression of SDC1 in the articular cartilage was upregulated, indicating that SDC1 participates in the repair of cartilage fibrils [51, 52].

In conclusion, the PPI network and data analyses are effective ways for screening OA target proteins and corresponding gene targets, which can further reveal the pathological and biological mechanisms of OA. In the present study, based on qRT-PCR results, *COL1A1*, *COL5A1*, *TIMP1*, *MMP13*, and *SDC1* may be gene diagnostic markers and drug targets of OA. Even though we validated several hub genes and pathways, the small sample size is one limitation of this study. Further large-scale experimental studies are needed to confirm these preliminary results.

## Figures and Tables

**Table 1:** The primers of the top 10 hub genes.

Gene	Forward primer	Reverse primer
FN1	AGGAAGCCGAGGTTTTAACTG	AGGACGCTCATAAGTGTCACC
COL1A1	GAGGGCCAAGACGAAGACATC	CAGATCACGTCATCGCACAAC
IGF1	GCTCTTCAGTTCGTGTGTGGA	GCCTCCTTAGATCACAGCTCC
SPP1	GAAGTTTCGCAGACCTGACAT	GTATGCACCATTCAACTCCTCG
TIMP1	CTTCTGCAATTCCGACCTCGT	ACGCTGGTATAAGGTGGTCTG
BGN	CAGTGGCTTTGAACCTGGAG	GGGAGGTCTTTGGGGATGC
COL5A1	GCCCGGATGTCGCTTACAG	AAATGCAGACGCAGGGTACAG
MMP13	ACTGAGAGGCTCCGAGAAATG	GAACCCCGCATCTTGGCTT
CLU	CCAATCAGGGAAGTAAGTACGTC	CTTGCGCTCTTCGTTTGTTTT
SDC1	CTGCCGCAAATTGTGGCTAC	TGAGCCGGAGAAGTTGTCAGA
GAPDH	CGGACCAATACGACCAAATCCG	AGCCACATCGCTCAGACACC

**Table 2:** The significantly enriched analysis of DEGs in OA.

Expression	Category	Term	Description	Gene count	*P* value
DEGs	BP BP BP BP BP CC CC CC CC CC MF MF MF MF MF	GO:0007155 GO:0030198 GO:0050870 GO:0001525 GO:0001649 GO:0005615 GO:0031012 GO:0070062 GO:0005576 GO:0005578 GO:0005518 GO:0005178 GO:0005201 GO:0016504 GO:0000977	Cell adhesion Extracellular matrix organization Positive regulation of T cell activation Angiogenesis Osteoblast differentiation Extracellular space Extracellular matrix Extracellular exosome Extracellular region Proteinaceous extracellular matrix Collagen binding Integrin binding Extracellular matrix structural constituent Peptidase activator activity RNA polymerase II regulatory region sequence-specific DNA binding	15 10 4 9 6 35 16 47 31 11 6 6 5 3 7	5.17*E*-05 5.76*E*-05 4.87*E*-04 7.83*E*-04 0.00220487 3.51*E*-09 2.52*E*-08 2.26*E*-06 1.95*E*-05 8.13*E*-05 1.79*E*-04 0.002314609 0.002834025 0.003280773 0.010113065

**Table 3:** Signaling pathway enrichment analysis of DEG function in OA.

Expression	Term	Description	Gene count	*P* value
DEGs	hsa04512	ECM-receptor interaction	6	0.001503576
	hsa04066	HIF-1 signaling pathway	5	0.013969316
	hsa04510	Focal adhesion	7	0.01467336
	hsa04152	AMPK signaling pathway	5	0.031444563
	hsa04380	Osteoclast differentiation	5	0.038325066
	hsa04910	Insulin signaling pathway	5	0.045003644
	hsa04145	Phagosome	5	0.057882463
	hsa05202	Transcriptional misregulation in cancer	5	0.079165687
	hsa01212	Fatty acid metabolism	3	0.079189222

## Data Availability

All data generated or analyzed during this study are included in this article.
